# RNautophagy/DNautophagy possesses selectivity for RNA/DNA substrates

**DOI:** 10.1093/nar/gkv579

**Published:** 2015-06-01

**Authors:** Katsunori Hase, Yuuki Fujiwara, Hisae Kikuchi, Shu Aizawa, Fumihiko Hakuno, Shin-Ichiro Takahashi, Keiji Wada, Tomohiro Kabuta

**Affiliations:** 1 Department of Degenerative Neurological Disease, National Institute of Neuroscience, National Center of Neurology and Psychiatry, 4-1-1 Ogawahigashi, Kodaira, Tokyo 187-8502, Japan; 2 Department of Animal Sciences and Applied Biological Chemistry, Graduate School of Agriculture and Life Sciences, The University of Tokyo, 1-1-1 Yayoi, Bunkyo-ku, Tokyo 113-8657, Japan; 3 Department of Electrical Engineering and Bioscience, Graduate School of Advanced Science and Engineering, Waseda University, 2-2 Wakamatsu-cho, Sinjuku-ku, Tokyo 162-8480, Japan

## Abstract

Lysosomes can degrade various biological macromolecules, including nucleic acids, proteins and lipids. Recently, we identified novel nucleic acid-degradation systems termed RNautophagy/DNautophagy (abbreviated as RDA), in which RNA and DNA are directly taken up by lysosomes in an ATP-dependent manner and degraded. We also found that a lysosomal membrane protein, LAMP2C, the cytoplasmic region of which binds to RNA and DNA, functions, at least in part, as an RNA/DNA receptor in the process of RDA. However, it has been unclear whether RDA possesses selectivity for RNA/DNA substrates and the RNA/DNA sequences that are recognized by LAMP2C have not been determined. In the present study, we found that the cytosolic region of LAMP2C binds to poly-G/dG, but not to poly-A/dA, poly-C/dC, poly-dT or poly-U. Consistent with this binding activity, poly-G/dG was transported into isolated lysosomes via RDA, while poly-A/dA, poly-C/dC, poly-dT and poly-U were not. GGGGGG or d(GGGG) sequences are essential for the interaction between poly-G/dG and LAMP2C. In addition to poly-G/dG, G/dG-rich sequences, such as a repeated GGGGCC sequence, interacted with the cytosolic region of LAMP2C. Our findings indicate that RDA does possess selectivity for RNA/DNA substrates and that at least some consecutive G/dG sequence(s) can mediate RDA.

## INTRODUCTION

Lysosomes contain various types of hydrolases, such as proteases, endonucleases, exonucleases and lipases, which are able to degrade virtually all types of biological macromolecules. Lysosomes contribute to the maintenance of cellular and biological homeostasis by keeping a balance between the synthesis and degradation of intracellular components (1,2). Deficiency of any of the lysosomal enzymes or proteins causes lysosomal storage disease in humans (3). Loss of DNase II, which is a lysosomal DNase, results in embryonic lethality in mice (4), while deficiency of RNase T2, which is an RNase localized in lysosomes (5), leads to leukoencephalopathy in humans (6). These observations indicate that degradation of RNA/DNA in lysosomes is essential for biological homeostasis. Therefore, it is important to elucidate the mechanisms by which nucleic acids are transported and degraded in lysosomes.

Intracellular systems that deliver cytosolic components into lysosomes/vacuoles for degradation are called autophagy (7). To date, at least three types of autophagy, macroautophagy, microautophagy and chaperone-mediated autophagy have been reported. Macroautophagy is characterized by an autophagosome, a double-membrane vesicle, which engulfs cytosolic constituents and delivers them to lysosomes via membrane trafficking (8). Microautophagy is an uptake and degradation process of cytosolic constituents involving invagination of the lysosomal membrane. However, microautophagy is poorly understood in mammals (9). In chaperone-mediated autophagy, substrate proteins in the cytosol are directly taken up by lysosomes via the chaperone protein, Hsc70, and a lysosomal membrane protein, LAMP2A (10,11), which is one of the proteins translated from three splice variants of *LAMP2, LAMP2A, LAMP2B* and *LAMP2C* (12).

In addition to these pathways, we recently identified a novel type(s) of autophagy, which we termed ‘RNautophagy’ and ‘DNautophagy’ (13,14). In this system(s), RNA/DNA is directly taken up by lysosomes in an adenosine triphosphate (ATP) dependent manner, and degraded. We also revealed that the cytosolic region of LAMP2C directly binds to nucleic acids, and that LAMP2C, at least in part, functions as a receptor for RNautophagy/DNautophagy (hereafter referred to as RDA). However, whether or not RDA possesses substrate-selectivity has remained unclear and the sequences that are recognized by LAMP2C have not been elucidated.

In the present study, to elucidate the RNA/DNA sequences that interact with LAMP2C, we performed binding assays using oligonucleotide-derived RNAs/DNAs containing various sequences. We also examined the relationship between the binding activity of RNAs/DNAs to LAMP2C and whether or not each RNA/DNA is translocated to lysosomes by RDA.

## MATERIALS AND METHODS

### Peptides and oligonucleotides

Biotinylated peptides were prepared as described previously (13–15). The sequences of the peptides were as follows. Control peptide: [Biotin]-GSGSGSGSGS; Human LAMP2C peptide: [Biotin]-GSGSGSGSGSIGRRKSRTGYQSV; Human LAMP2A peptide: [Biotin]-GSGSGSGSGSIGLKHHHAGYEQF. All oligonucleotides were synthesized by FASMAC Co.

### Pull-down assay

Pull-down assays were performed as previously described (13,14). Streptavidin Sepharose High Performance beads (GE Healthcare) were blocked overnight with 3% bovine serum albumin in Dulbecco's phosphate-buffered saline (PBS) at 4°C. The blocked beads were incubated at 4°C for 2 h with 1 or 2 nmol of RNA/DNA and 8 nmol of biotin conjugated peptides in PBS containing 0.05% Triton-X 100. After incubation, beads were washed three times with 0.05% Triton-X 100 in PBS or with PBS. Pulled down RNA was extracted using TRIzol (Life Technologies). For extraction of pulled-down DNA, beads were incubated for 120 min at 37°C in DNA extraction buffer (10 mM Tris–HCl, pH 7.5, 1 mM ethylene-diaminetetraacetic acid, pH 8.0, 1% sodium dodecyl sulfate, 0.1 mg/ml Proteinase K in saline-sodium citrate buffer), followed by phenol–chloroform extraction. Extracted DNAs and RNAs were analyzed by agarose gel electrophoresis, or quantified by measuring their OD_260_. Input indicates 100% input unless otherwise mentioned.

### Uptake of DNA and RNA by isolated lysosomes

Isolation of lysosomes and uptake assays using isolated lysosomes were performed as previously described (13,14). Briefly, lysosomes were isolated from 10–12-week-old whole mouse brains using a Lysosome Enrichment Kit (Thermo Scientific). Lysosomes isolated by this procedure contain minimal contamination with other organelles (14). Isolated lysosomes (5–10 μg of protein) and 100 or 200 pmol of RNA/DNA were incubated in 30 μl of 0.3 M sucrose containing 10 mM MOPS buffer (pH 7.0) with or without energy regenerating system (10 mM ATP, 10 mM MgCl_2_, 2 mM phosphocreatine and 50 μg/ml creatine phosphokinase) at 37°C. After incubation for 5 min, lysosomes were removed by centrifugation and RNA was extracted from the assay solution outside of lysosomes using TRIzol. For extraction of DNA, the assay solution outside of lysosomes was incubated for 120 min at 37°C with DNA extraction buffer, followed by phenol–chloroform extraction. The levels of RNA/DNA remaining in the assay solution outside of lysosomes were detected by agarose gel electrophoresis and quantified using FluorChem (Alpha Innotech) and FluorChem software (Alpha Innotech). ssRNA and ssDNA were detected by annealing with complementary DNA before agarose gel electrophoresis. Input indicates 100% input. All animal experiments were approved by the animal experimentation committee of the National Center of Neurology and Psychiatry.

### Electron microscopy

Electron microscopy was performed as previously described with some modifications (13,14). Isolated lysosomes (50–100 μg of protein) and 0 or 2 nmol of RNA (poly-G_(15)_) were incubated with energy regenerating system at 37°C for 5 min, precipitated by centrifugation and fixed in 0.1% glutaraldehyde, 4% paraformaldehyde in phosphate buffer (pH 7.2) at 4°C overnight. Fixed lysosomes were then dehydrated in a series of water/ethanol mixtures to 100% ethanol and embedded in LR White (Nisshin EM Co.). The embedded samples were sectioned at 100 nm, collected on 400-mesh nickel grids and incubated with 20 μM biotinylated oligonucleotide DNA probe ([Biotin]-AAAAAAAAAAAAAAACCCCCCCCCCCCCCC) at room temperature overnight. Immunogold labeling was performed using streptavidin coupled with 10 nm gold particles (Sigma). Samples were viewed using a Tecnai Spirit transmission electron microscope (FEI) at 80 kV.

### Methylation of DNA

DNA was methylated using a CpG methyltransferase (M.SssI) (NEB) according to the manufacturer's instructions. Briefly, 4 nmol of dsDNA was incubated with S-adenosylmethionine (NEB) and CpG methyltransferase in NE Buffer (NEB) at 37°C. After 8 h of incubation, methylated DNA was purified using phenol-chloroform, followed by ethanol precipitation.

### Statistical analyses

For comparisons between two groups, statistical analyses were performed using Student's t-test. For comparison of more than two groups, Tukey's test was used.

## RESULTS

### LAMP2C interacts with poly-G/dG

**Figure 1.**
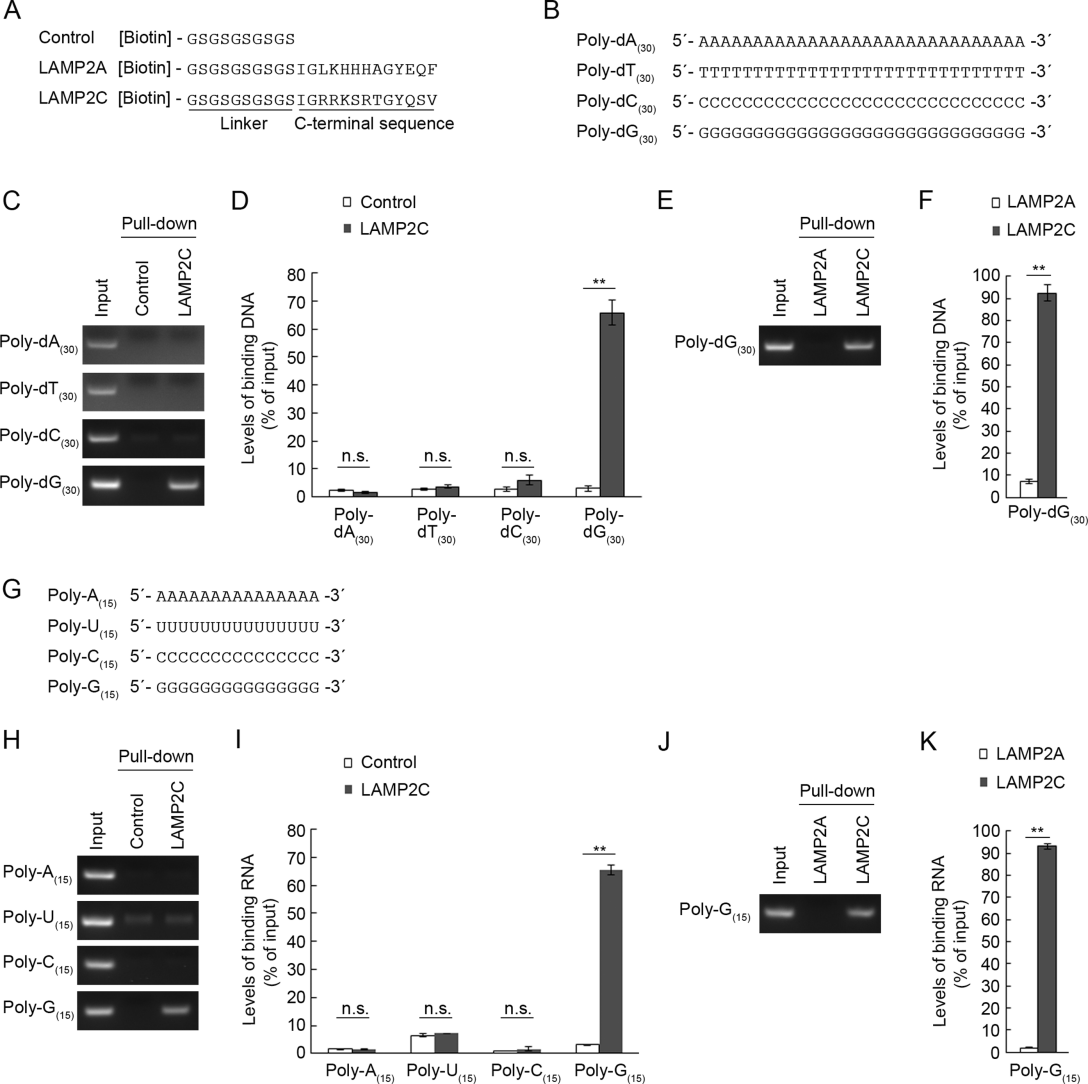
Interaction of poly-dG/G with the cytoplasmic sequence of LAMP2C. (**A**) A schematic representation of peptide sequences used for pull-down assays. (**B**) Oligonucleotide DNA sequences used for pull-down assays. (**C**–**F**) Interaction of the cytosolic sequence of LAMP2C with poly-dA_(30)_, poly-dT_(30)_, poly-dC_(30)_ and poly-dG_(30)_. Pull-down assays were performed using oligo DNA (1 nmol) (B) and biotinylated peptide (8 nmol) (A). After elution, ssDNA were annealed with complementary oligonucleotide DNA, and levels of bound DNA were analyzed by agarose gel electrophoresis, followed by EtBr staining (C and E). Relative levels of bound DNA were quantified by OD_260_ measurement (D and F). Mean values are shown with SEM (D: *n* = 4, F: *n* = 3). **P < 0.01, n.s., not significant (Tukey's test) (D). **P < 0.01 (*t*-test) (F). (**G**) Oligonucleotide RNA sequences used for pull-down assays. (**H**–**K**) Interaction of the cytosolic sequence of LAMP2C with poly-A_(15)_, poly-U_(15)_, poly-C_(15)_ and poly-G_(15)_. Pull-down assays were performed using oligo RNA (2 nmol) (G) and the biotinylated peptide (8 nmol). After elution, ssRNA were annealed with complementary oligonucleotide DNA, and levels of bound RNA were analyzed by agarose gel electrophoresis, followed by EtBr staining (H and J). Relative levels of bound RNA were quantified by OD_260_ measurement (I and K). Mean values are shown with SEM (*n* = 3). **P < 0.01, n.s., not significant (Tukey's test) (I). **P < 0.01 (*t*-test) (K).

The cytoplasmic region of LAMP2C consists of 11–12 amino acid residues and we have previously shown that a peptide construct of this sequence (Figure 1A) is useful for monitoring its interactions with RNA/DNA (13,14). In the present study, we used the peptide and RNAs/DNAs composed of short oligonucleotides to identify the nucleotide sequences that mediate RDA. To investigate which types of nucleotide in DNA preferentially interact with the cytoplasmic sequence of LAMP2C, we first prepared four 30-base ssDNAs, namely, poly-dA_(30)_, poly-dT_(30)_, poly-dC_(30)_, and poly-dG_(30)_ (Figure 1B) and performed pull-down assays using these oligonucleotides and the LAMP2C peptide. We found that poly-dG_(30)_ bound to the LAMP2C peptide, while the other ssDNAs, poly-dA_(30)_, poly-dT_(30)_ and poly-dC_(30)_ did not (Figure 1C and D).

The cytoplasmic sequence of LAMP2C contains the same number of amino acids as that of LAMP2A. We have previously shown that a peptide construct of the cytosolic sequence of human LAMP2A does not interact with RNA/DNA (13,14). The interaction between poly-dG_(30)_ and the LAMP2C peptide was also observed when we used the LAMP2A peptide as a negative control (Figure 1E and F), indicating that this interaction does not result from non-specific binding.

We next tested interaction between ssRNA and LAMP2C peptide, using four types of 15 base ssRNA, poly-A_(15)_, poly-U_(15)_, poly-C_(15)_ and poly-G_(15)_ (Figure 1G). The cytosolic sequence of LAMP2C clearly bound to poly-G_(15)_, but not to poly-A_(15)_, poly-U_(15)_ or poly-C_(15)_ (Figure 1H and I). We confirmed that the interaction between poly-G_(15)_ and the LAMP2C peptide was observed when we used the LAMP2A peptide as a negative control (Figure 1J and K).

**Figure 2.**
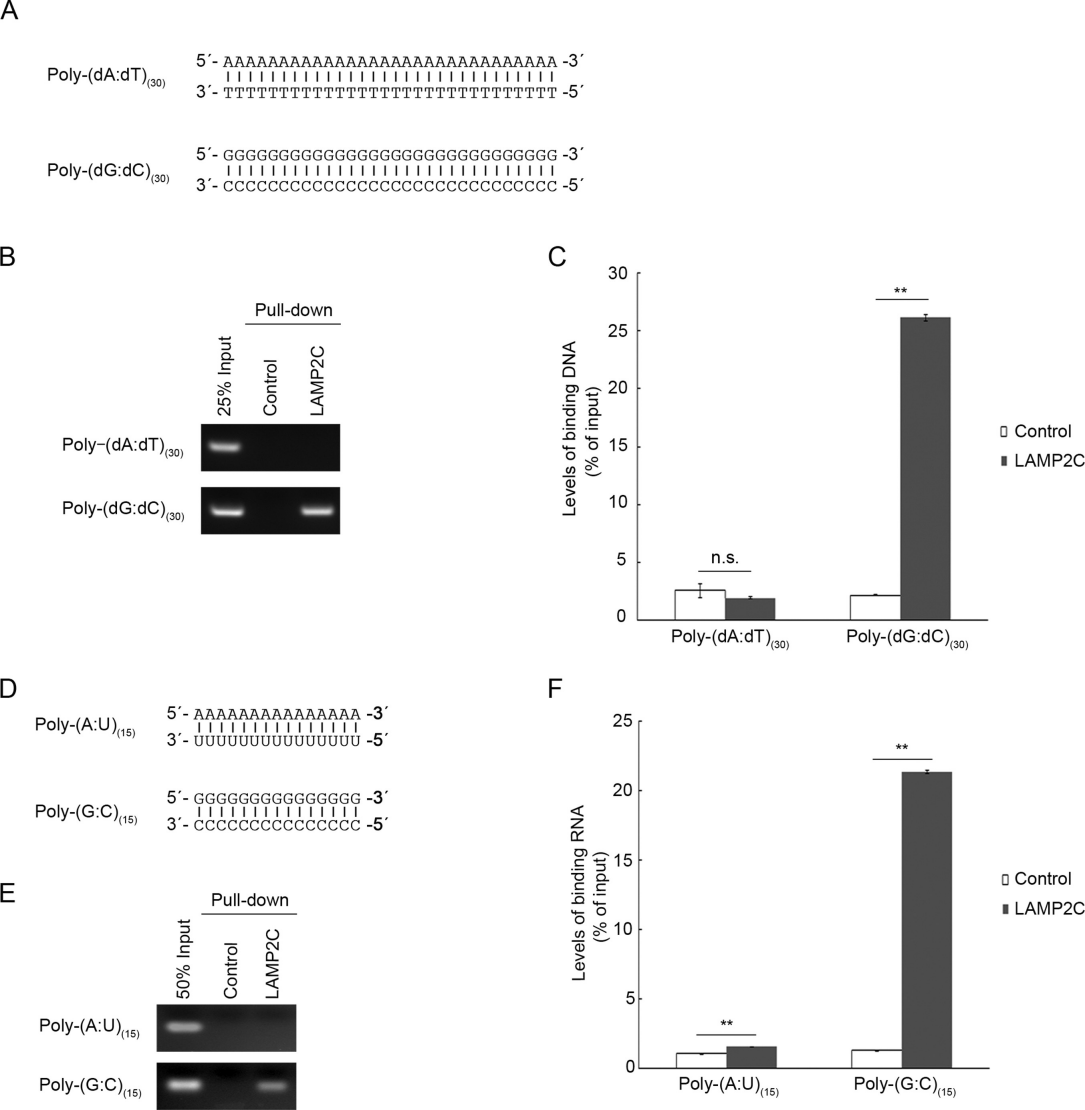
Interaction of poly-(dG:dC)/(G:C) with the cytoplasmic sequence of LAMP2C. (**A**) DsDNA sequences used for pull-down assays. (**B** and **C**) Interaction of the cytosolic sequence of LAMP2C with poly-(dA:dT)_(30)_ and poly-(dG:dC)_(30)_. Pull-down assays were performed using DNA (1 nmol) (A) and biotinylated peptide. The DNA bound to the cytosolic sequence of LAMP2C was assessed by electrophoresis in agarose gels, followed by EtBr staining (B). Relative levels of bound DNA were quantified by OD_260_ measurement (C). Mean values are shown with SEM (*n* = 3). **P < 0.01, n.s., not significant (Tukey's test). (**D**) DsRNA sequences used for pull-down assays. (**E** and **F**) Interaction of the cytosolic sequence of LAMP2C with poly-(A:U)_(15)_ and poly-(G:C)_(15)_. Pull-down assays were performed using RNA (2 nmol) (D). The RNA bound to the cytosolic sequence of LAMP2C was assessed by electrophoresis in agarose gels, followed by EtBr staining (E). Relative levels of bound RNA were quantified by OD_260_ measurement (F). Mean values are shown with SEM (*n* = 3). **P < 0.01 (Tukey's test).

We also examined interaction of LAMP2C with dsDNA/dsRNA. We performed a pull-down assay using poly-(dA:dT)_(30)_ and poly-(dG:dC)_(30)_ (Figure 2A). Consistent with the binding activity of ssDNA, LAMP2C peptide interacted with poly-(dG:dC)_(30)_, but not with poly-(dA:dT)_(30)_ (Figure 2B and C). Similarly, poly-(G:C)_(15)_ bound to LAMP2C peptide (Figure 2D–F). Although the levels of poly-(A:U)_(15)_ quantified by OD_260_ measurement were slightly higher in the samples pulled down with the LAMP2C peptide compared with control samples (Figure 2F), interaction between poly-(A:U)_(15)_ and the LAMP2C peptide was not detected by agarose gel analysis (Figure 2E), suggesting that poly-(A:U)_(15)_ does not bind to LAMP2C.

### RNA/DNA that contains poly-G/dG is taken up by isolated lysosomes

**Figure 3.**
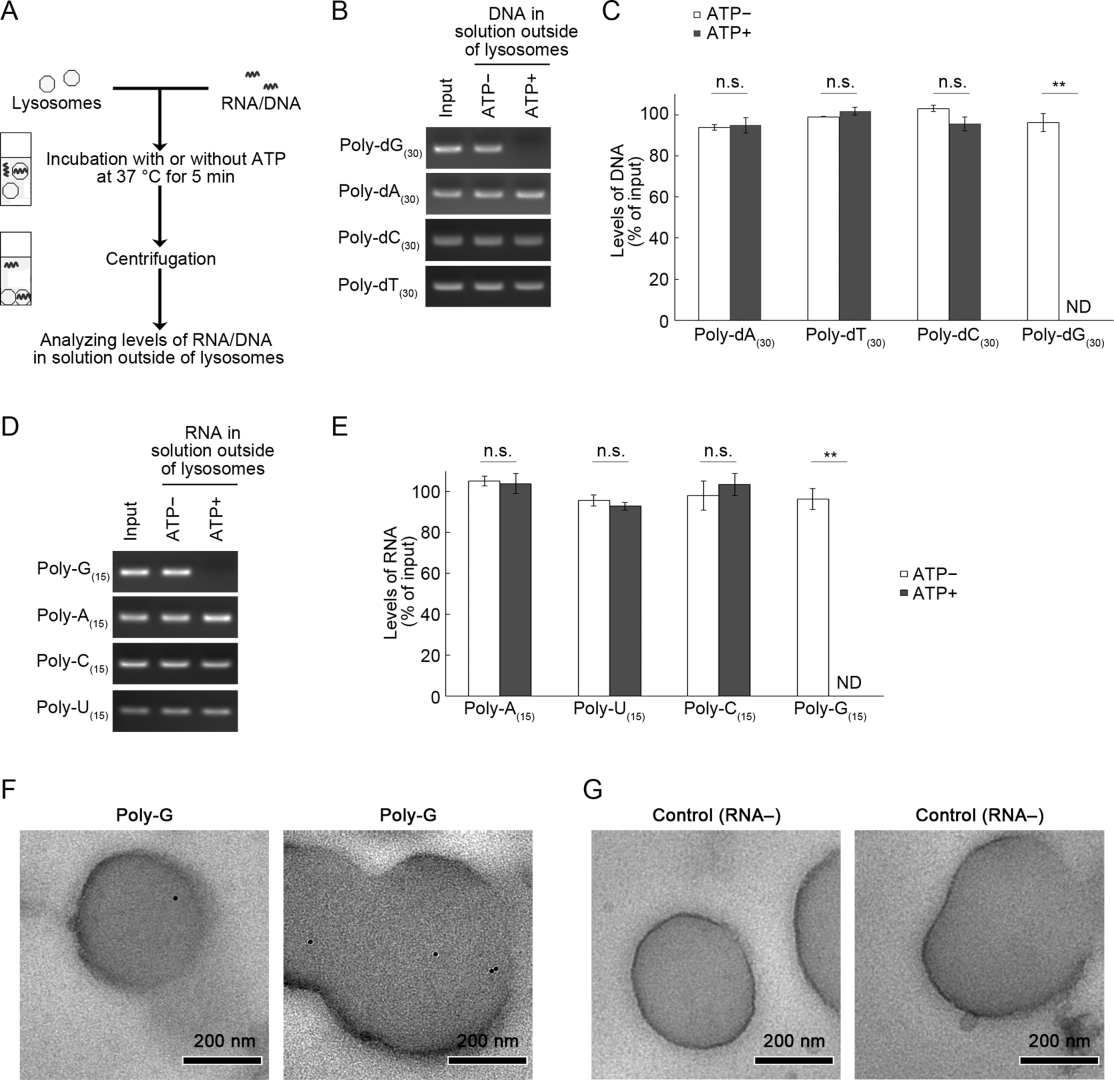
Uptake of poly-G/dG by isolated lysosomes. (**A**) Outline of RNA/DNA uptake assays using isolated lysosomes. (**B** and **C**) Poly-dA_(30)_, poly-dT_(30)_, poly-dC_(30)_ or poly-dG_(30)_ (100 pmol) were incubated for 5 min with lysosomes isolated from mouse brain in the presence or absence of an ATP regeneration system (ATP+, ATP−, respectively). After the incubation, ssDNA remaining in the solution outside of lysosomes or input ssDNA were annealed with complementary oligonucleotide DNA and levels of DNA were analyzed by agarose gel electrophoresis (B). Relative DNA levels shown in (B) were quantified by densitometry (C). Mean values are shown with SEM (*n* = 3). **P < 0.01, n.s., not significant (Tukey's test). (**D** and **E**) Uptake of poly-A_(15)_, poly-U_(15)_, poly-C_(15)_ and poly-G_(15)_ (200 pmol) were incubated for 5 min with isolated lysosomes in the presence or absence of an ATP regeneration system. After the incubation, ssRNA remaining in the solution outside of lysosomes or input ssRNA were annealed with complementary oligonucleotide DNA and levels of RNA (RNA–DNA hybrid) were analyzed by agarose gel electrophoresis (B). Relative RNA levels shown in (B) were quantified by densitometry (C). Mean values are shown with SEM (*n* = 3). **P < 0.01, n.s., not significant (Tukey's test). ND, not detected. (**F** and **G**) Isolated lysosomes were incubated with poly-G_(15)_ (F) or without RNA (G) in the presence of an ATP regeneration system. Poly-G was detected by post-embedding electron microscopy using biotinylated oligonucleotide DNA probe containing poly-C sequence and streptavidin-colloidal gold (10 nm). Gold particles were observed in the lysosomes incubated with poly-G (F). No gold particles were observed in the lysosomes incubated without RNA (G).

To analyze the correlation between the uptake of RNA/DNA by lysosomes and the binding abilities of RNA/DNA with LAMP2C, we performed cell-free lysosomal uptake assays according to the method we have previously reported (13,14). In the RNA/DNA uptake assays, isolated lysosomes and RNA/DNA (in the form of oligonucleotides) were mixed and incubated for 5 min at 37°C in the presence or absence of ATP (energy regenerating system). Lysosomes were then removed by centrifugation and the levels of RNA/DNA remaining in the uptake assay solution (i.e. not taken up by lysosomes) were analyzed by agarose gel electrophoresis (Figure 3A). Because RDA functions in an ATP-dependent manner, by comparing levels of RNA/DNA remaining in ATP+ samples with those in ATP− samples, we can assess whether specific RNAs/DNAs are transported into isolated lysosomes. We have observed that nonspecific degradation of RNA did not occur in the assay solution outside of lysosomes (Supplementary Figure S1), while RNA was degraded by detergent-lysed lysosomes (Supplementary Figure S2), indicating that isolated lysosomes are intact. We have previously confirmed, using post-embedding immunoelectron microscopy analyses, that RNA/DNA is translocated into the lysosomal lumen in our experimental conditions (13,14).

Lysosomal uptake assays showed that poly-dG_(30)_ was transported into isolated lysosomes in the presence of ATP (Figure 3B and C). In contrast, poly-dA_(30)_, poly-dT_(30)_ and poly-dC_(30)_ were not translocated into lysosomes even in the presence of ATP (Figure 3B and C). We confirmed that in isolated lysosomes incubated without RNA/DNA, RNA/DNA was not detected by agarose electrophoresis with ethidium bromide staining (Supplementary Figure S3). We also confirmed that poly-dG_(30)_ was not degraded in the solution outside of lysosomes (Supplementary Figure S4). We observed that double-stranded poly-(dG:dC)_(30)_ was transported into isolated lysosomes, but that poly-(dA:dT)_(30)_ was not (Supplementary Figure S5). Similarly, poly-G_(15)_ was translocated into lysosomes by RNautophagy, but poly-A_(15)_, poly-U_(15)_ and poly-dC_(15)_ were not (Figure 3D and E). For further confirmation of the translocation of poly-G into lysosomes, poly-G_(15)_ was incubated with isolated lysosomes in the presence of ATP and localization of poly-G was visualized by post-embedding electron microscopy using biotinylated oligonucleotide DNA containing poly-C sequence and streptavidin-colloidal gold. As a result, poly-G was clearly detected inside lysosomes (Figure 3F and G).

Overall, poly-G/dG-containing RNAs/DNAs could bind to LAMP2C and were substrates of RDA, while other RNAs/DNAs that do not have binding activity to LAMP2C were not. These results indicate that RDA is not a nonspecific degradation system and that RDA possesses selectivity for RNA/DNA substrates.

### GGGGGG or d(GGGG) sequence is essential for the interaction between poly-G/dG and LAMP2C

**Figure 4.**
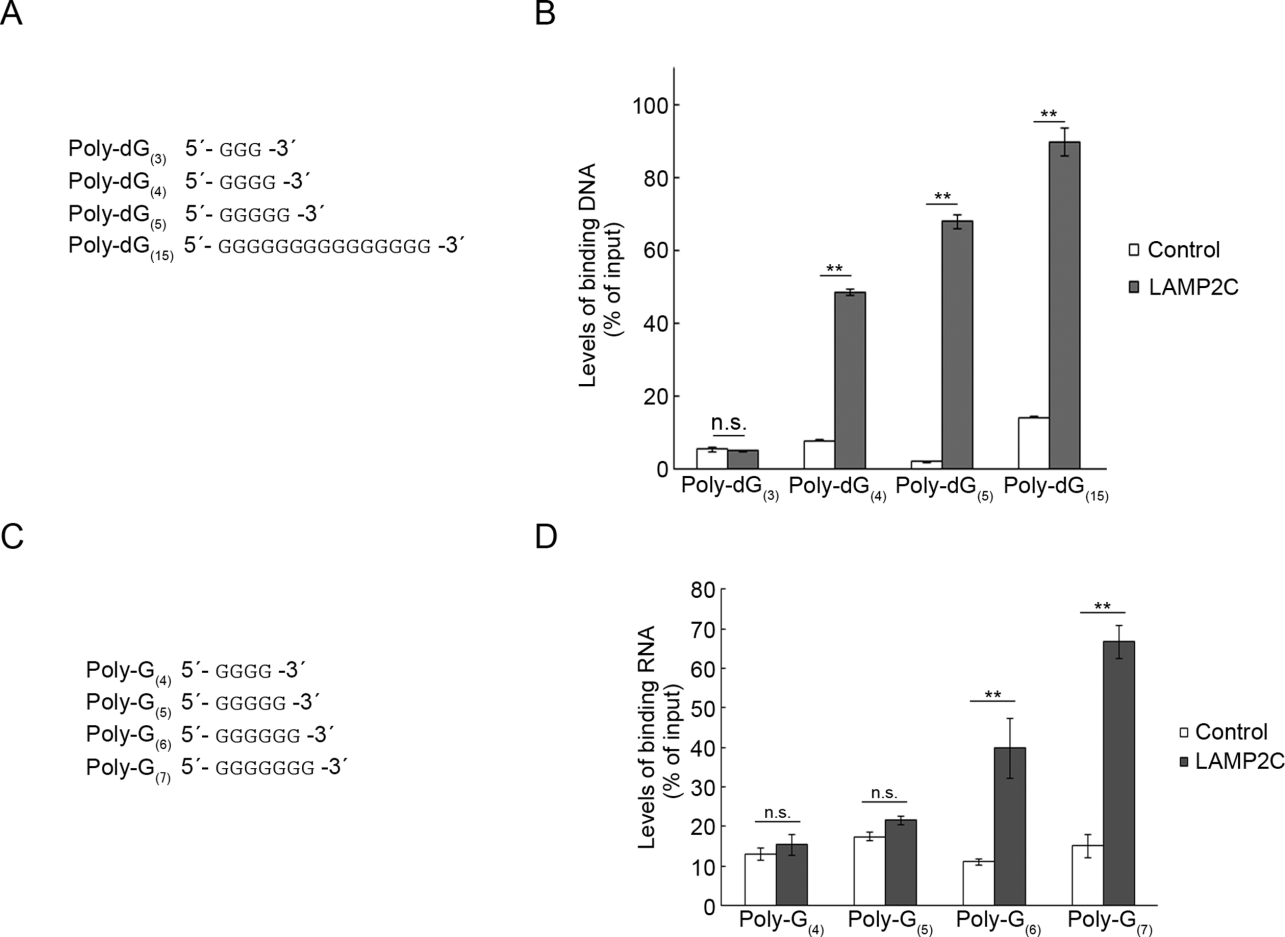
Minimal poly-dG/G sequences that are required for interaction with the cytoplasmic sequence of LAMP2C. (**A**) Poly-dG sequences used for pull-down assays. (**B**) Pull-down assays were performed using DNAs shown in (A). Relative levels of bound DNA were quantified by OD_260_ measurement. Mean values are shown with SEM (n = 3). **P < 0.01, n.s., not significant (Tukey's test). (**C**) Poly-G sequences used for pull-down assays. (**D**) Pull-down assays were performed using RNAs shown in (C). Relative levels of bound RNA were quantified by OD_260_ measurement. Mean values are shown with SEM (n = 3). **P < 0.01, n.s., not significant (Tukey's test).

We next examined the interaction between LAMP2C peptide and various lengths of poly-dG (poly-dG_(3)_, poly-dG_(4)_, poly-dG_(5)_ and poly-dG_(15)_) (Figure 4A). Poly-dGs consisting of more than three nucleotides interacted with the LAMP2C peptide, while poly-dG_(3)_ did not (Figure 4B), indicating that a d(GGGG) sequence is essential and sufficient for the interaction between poly-dG and the LAMP2C peptide.

We also assessed the length of poly-G RNA that is required for interaction with the LAMP2C peptide using various poly-Gs (poly-G_(4)_, poly-G_(5)_, poly-G_(6)_ and poly-G_(7)_) (Figure 4C). Poly-G_(6)_ and poly-G_(7)_ interacted with the LAMP2C peptide (Figure 4D), although poly-G_(4)_ and poly-G_(5)_ did not, indicating that a GGGGGG sequence is essential and sufficient for the interaction between poly-G and the LAMP2C peptide.

### Multiple d(GGGG) sequences in dsDNA and GGGG sequences in RNA interact with LAMP2C

**Figure 5.**
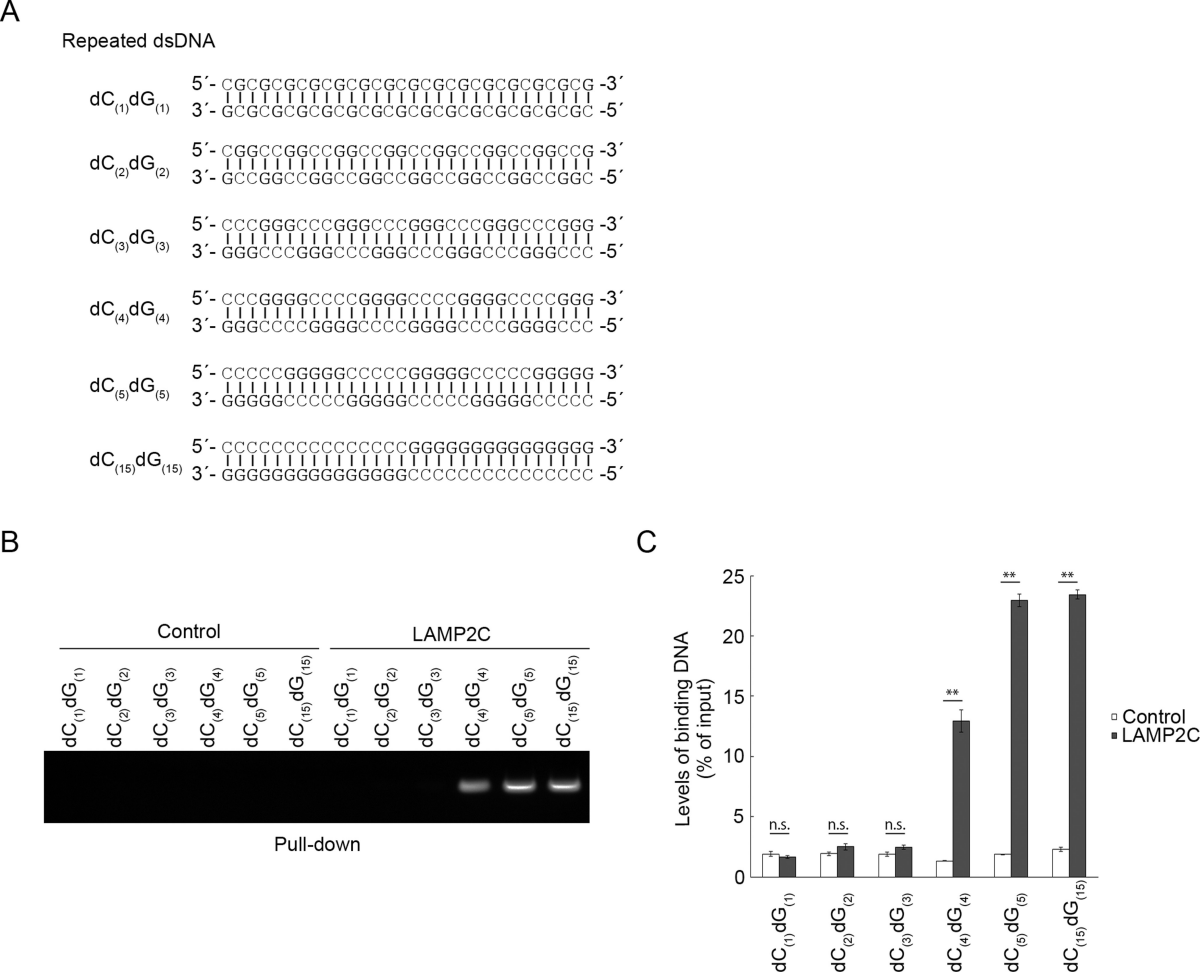
DNA repeat sequences that interact with LAMP2C. (**A**) DsDNA sequences used for pull-down assays. (**B** and **C**) Pull-down assays were performed using DNAs shown in (A). The DNA bound to the cytosolic sequence of LAMP2C was assessed by electrophoresis in agarose gels, followed by EtBr staining (B). Relative levels of bound DNA were quantified by OD_260_ measurement (C). Mean values are shown with SEM (*n* = 3). **P < 0.01, n.s., not significant (Tukey's test).

We next tested whether or not G/dG-rich sequences also contribute to substrate-recognition in RN/DNautophagy. We used dsDNAs that consist of repeated d(CG), d(CCGG), d(CCCGGG), d(CCCCGGGG) or d(CCCCCGGGGG) sequences or dsDNA consisting of poly-dC_(15)_-dG_(15)_ (Figure 5A). We observed that dsDNAs with repeated d(CCCCGGGG), d(CCCCCGGGGG) or poly-dC_(15)_-dG_(15)_ sequences exhibited binding activity to the LAMP2C peptide (Figure 5B and C). dsDNAs consisting of repeated d(CG), d(CCGG) or d(CCCGGG) sequences did not bind to the LAMP2C peptide (Figure 5B and C). These results are consistent with our observation that a d(GGGG) sequence is essential and sufficient for the interaction between poly-dG and the LAMP2C peptide (Figure 4).

**Figure 6.**
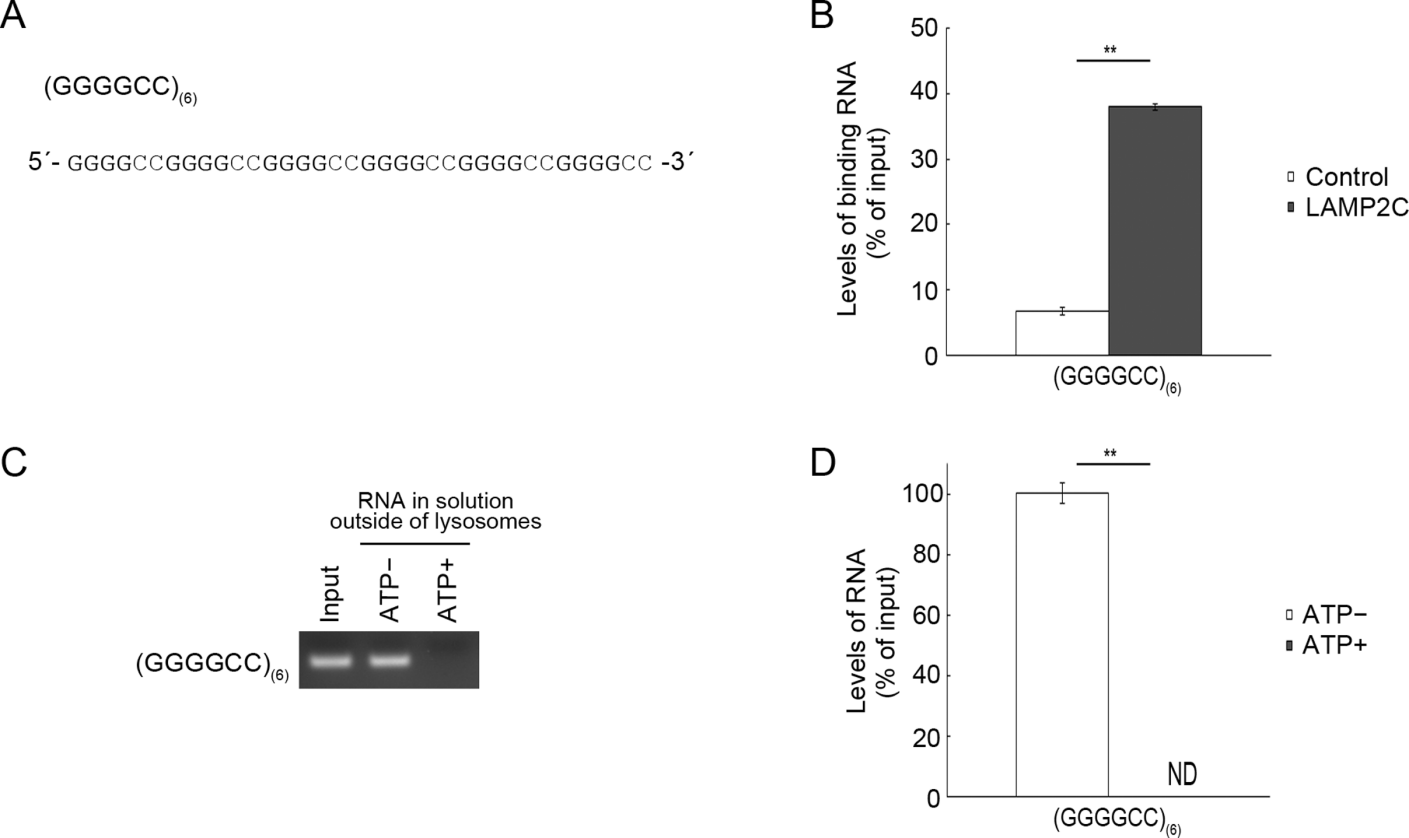
RNA repeat sequences that interact with LAMP2C and mediate RDA. (**A**) RNA sequences used for pull-down assays and uptake assays. (**B**) Pull-down assays were performed using RNAs shown in (A). Relative levels of bound RNA were quantified by OD_260_ measurement. Mean values are shown with SEM (*n* = 3). **P < 0.01 (*t*-test). (**C** and **D**) Uptake of RNAs shown in (A) by isolated lysosomes was examined. Mean values are shown with SEM (*n* = 3). **P < 0.01 (*t*-test).

We then examined an RNA containing multiple GGGG sequences. We used an RNA consisting of a repeated GGGGCC sequence (Figure 6A). While poly-G_(4)_ does not interact with the LAMP2C peptide, as shown above (Figure 4), RNA consisting of repeated GGGGCC sequences interacted with the cytoplasmic sequence of LAMP2C (Figure 6B) and was transported into isolated lysosomes (Figure 6C and D). These results indicate that, in addition to a GGGGGG sequence, multiple GGGG sequences in RNA are capable of binding to the cytoplasmic sequence of LAMP2C and mediating RNautophagy.

### DNA methylation does not affect interaction between LAMP2C and DNA

**Figure 7.**
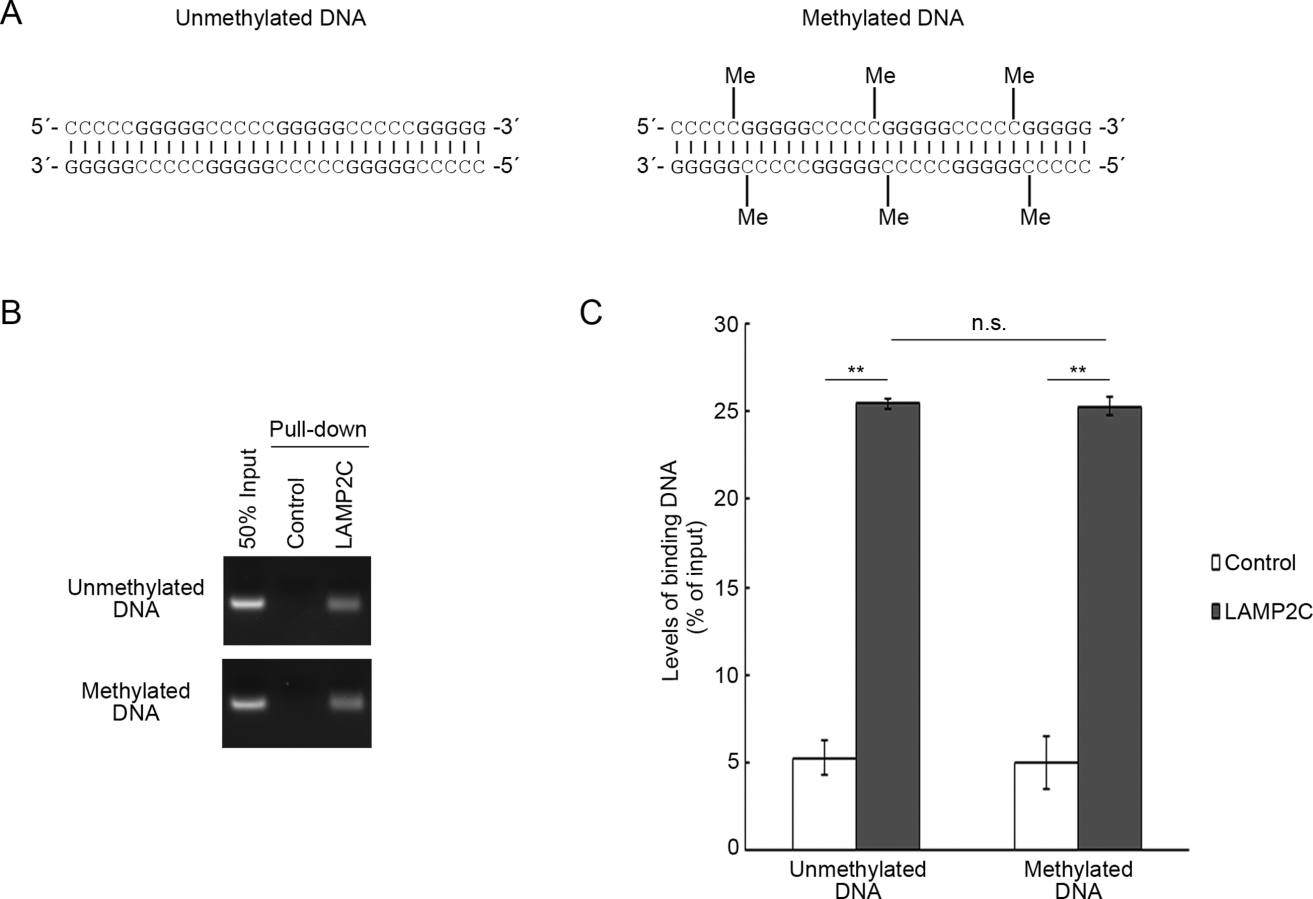
Effect of DNA methylation on its interaction with the cytosolic sequence of LAMP2C. (**A**) A Schematic representation of methylated and unmethylated DNA sequence used for pull-down assays. (**B** and **C**) Pull-down assays were performed using methylated and unmethylated DNA. The DNA bound to the cytosolic sequence of LAMP2C was assessed by electrophoresis in agarose gels, followed by EtBr staining (B). Relative levels of bound DNA were quantified by OD_260_ measurement (C). Mean values are shown with SEM (*n* = 3). **P < 0.01, n.s., not significant (Tukey's test).

Finally, we investigated the effect of DNA modification on the interaction between LAMP2C and DNA. We tested whether cytosine methylation, one of the major DNA modifications, affects the interaction because cytosine pairs with guanine. We performed a pull-down assay using methylated DNA (Figure 7A). However, DNA methylation did not affect the interaction levels between DNA and the LAMP2C peptide (Figure 7B and C).

## DISCUSSION

In the present study, we found that the cytosolic sequence of LAMP2C binds to poly-G/dG, but not to poly-A/dA, poly-C/dC, poly-dT or poly-U. Poly-G/dG was taken up by isolated lysosomes, but poly-A/dA, poly-C/dC, poly-dT and poly-U were not substrates of RDA. We also identified several minimal sequences that are able to bind to the cytoplasmic sequence of LAMP2C: GGGGGG in ssRNA and d(GGGG) in ssDNA. Our results indicate that RDA possesses substrate-selectivity at the step of nucleic acid uptake into lysosomes and that RDA is not a non-specific degradation system.

In cells, we assume that RNautophagy is also substrate-selective at the step of nucleic acid transport from cytoplasm or nucleus to lysosomes because RNA degradation is generally a controlled process in cells (16). We predict that RNA-binding proteins regulate the transport of RNA to the lysosomal membrane.

We have previously reported that LAMP2C functions as an RNA/DNA receptor in the process of RDA, but that RDA was not completely abolished in lysosomes from LAMP2 KO mice, suggesting the existence of LAMP2-independent RDA pathway(s) (13,14). Importantly, RNAs/DNAs that can bind to the LAMP2C peptide were all taken up by lysosomes, while others were not (Figures 1–6). These results strongly suggest that there is a LAMP2C-like RNA/DNA receptor(s) that functions in RDA, and that the substrate-selectivity at the step of lysosomal uptake is determined by the binding ability of substrates to RNA/DNA receptor(s) on the lysosomal membrane. Our findings indicate that RNA/DNA receptor(s) play a critical role in RDA and that this pathway is a controlled system. We have reported that LAMP2B, LAMP1 and LAMP4/CD68 are also able to bind to RNA/DNA (17). These LAMP family proteins are potential candidates as RNA/DNA receptors in the process of RDA.

An RNA consisting of repeated GGGGCC interacted with the cytoplasmic sequence of LAMP2C and was substrates of RDA, despite not containing GGGGGG sequences. These results suggest that LAMP2C interacts with particular nucleic acid structures. G/dG-rich sequences, including sequences containing multiple GGGG/d(GGGG) sequences, can form both intramolecular and intermolecular four-stranded structures called G-quadruplexes, (18–20). Repeated GGGGCC has been reported to form G-quadruplex (21). Therefore, it is possible that G-quadruplexes are capable of binding to the cytoplasmic sequence of LAMP2C and mediating RDA. Besides G-quadruplex, nucleic acids are able to adopt many structures, such as pseudoknot and hairpin. Whether LAMP2C interacts with particular nucleic acid structures is an interesting issue to be resolved.

Although we identified in this study consecutive G/dG sequences as a motif that interacts with LAMP2C, we predict that multiple additional sequences may interact with the cytoplasmic sequence of LAMP2C and mediate RDA. Further studies are needed to resolve this point.

Recently, we reported that the cytoplasmic sequence of LAMP2C binds to nucleic acids via an arginine-rich motif (17), which is a common RNA-binding motif. The specific binding of LAMP2C to guanine-containing nucleic acids shown in the present study is also reasonable from this point of view, because in many cases, arginine residues in arginine-rich motifs form hydrogen bonds with the major groove edge of guanine in both RNA and DNA (22–26). However, the arginine-rich motif of LAMP2C could also bind to nucleic acid sequences devoid of guanine residues because arginine residues can also form hydrogen bonds with the phosphate backbone and, in some cases, with bases other than guanine (22–26).

In mammals, about 60–90% of CpG sites are methylated, while viral, bacterial and mitochondrial DNA (mtDNA) have mostly unmethylated CpG sites (27). We previously reported that mtDNA is a substrate of DNautophagy, at least *in vitro* (13). In the present study, we have shown that DNA methylation does not affect the interaction between LAMP2C and DNA (Figure 7), suggesting that, at the step of lysosomal uptake, DNautophagy does not distinguish between self and non-self DNAs. Considering that DNA does not usually exist in the cytoplasm, and that mitochondrial and viral DNA are released into the cytoplasm under certain conditions (28,29), the possible substrates of DNautophagy may be mitochondrial DNA and viral DNA, and it is probably not important for DNautophagy to distinguish methylated and unmethylated DNA at the step of lysosomal uptake.

We have shown that RNA consisting of repeated GGGGCC sequences is a substrate of RNautophagy (Figure 6). Recent studies have shown that expanded d(GGGGCC) repeats between exons 1a and 1b in *C9ORF72* cause amyotrophic lateral sclerosis (ALS) associated with frontotemporal dementia (30,31). ALS is a fatal human neurodegenerative disease affecting primarily motor neurons (32,33). In the pathology of *C9ORF72*-associated ALS, pre-mRNA containing expanded GGGGCC repeats is thought to be toxic for neurons (34,35). We have previously reported that LAMP2C is highly expressed in neurons. These observations, together with our present findings, indicate that RNautophagy may be involved in the degradation of pre-mRNA containing expanded GGGGCC repeats in this disease.

Considering that RDA is substrate-selective, we presume that RDA contributes to turnover of endogenous RNA and degradation of exogenous RNA/DNA and toxic RNA such as disease-associated repeated RNA in cells. The biological significance of RDA is currently the subject of ongoing research.

## SUPPLEMENTARY DATA

Supplementary Data are available at NAR Online.

SUPPLEMENTARY DATA
