# Supplementary material for: RNautophagy/DNautophagy possesses selectivity for RNA/DNA substrates
Source: Nucleic Acids Res. 2015 Jun 1;43(13):6439–49. doi: 10.1093/nar/gkv579 (PMC4513860; doi:10.1093/nar/gkv579)
Supplement: SUPPLEMENTARY DATA [file supp_43_13_6439__index.html]

RNautophagy/DNautophagy possesses selectivity for RNA/DNA substrates — RNautophagy/DNautophagy possesses selectivity for RNA/DNA substrates — SUPPLEMENTARY DATA 

# RNautophagy/DNautophagy possesses selectivity for RNA/DNA substrates

## SUPPLEMENTARY DATA

- SUPPLEMENTARY DATA
