# Supplementary material for: RNautophagy/DNautophagy possesses selectivity for RNA/DNA substrates
Source: Nucleic Acids Res. 2015 Jun 1;43(13):6439–49. doi: 10.1093/nar/gkv579 (PMC4513860; doi:10.1093/nar/gkv579)
Supplement: SUPPLEMENTARY DATA [file supp_gkv579_nar-00759-y-2015-File009.pdf]

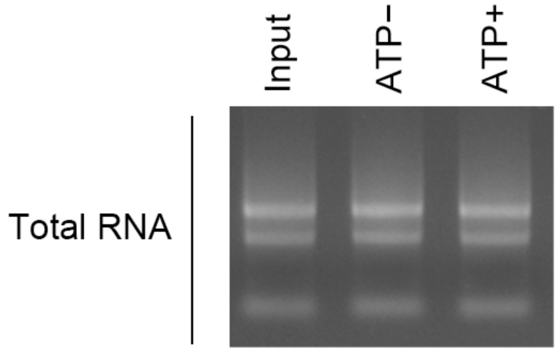

Figure S1. RNA was not degraded in the solution outside lysosomes. Isolated lysosomes were incubated for 5 min at 37°C in the presence or absence of an ATP regeneration system. The lysosomes were removed by centrifugation, and the solution outside lysosomes was incubated with 1 μg of purified total RNA derived from mice brains for 5 min at 37°C.

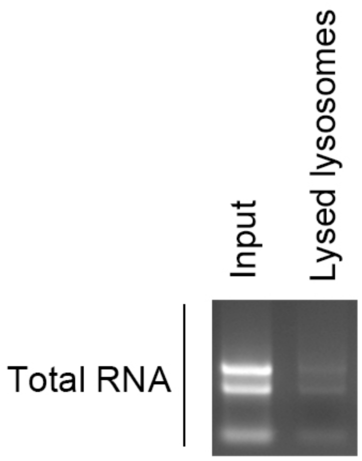

Figure S2. Isolated lysosomes were lysed in citrate-phosphate buffer (pH 5.0) containing 1% Triton X-100, mixed with 1 μg of purified total RNA, and incubated for 5 min at 37°C.

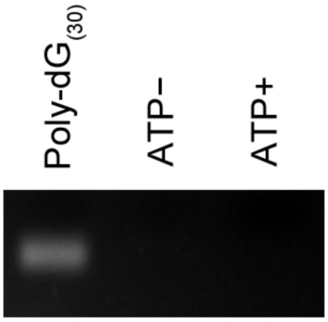

Figure S3. Isolated lysosomes were incubated for 5 min at 37°C without RNA/DNA in the presence or absence of an ATP regeneration system (ATP+, ATP−, respectively). After the incubation, phenol-chloroform extraction was performed, and levels of nucleic acids in the samples were analyzed by agarose electrophoresis with EtBr staining. Detectable nucleic acids were not observed in ATP+ and ATP− samples.

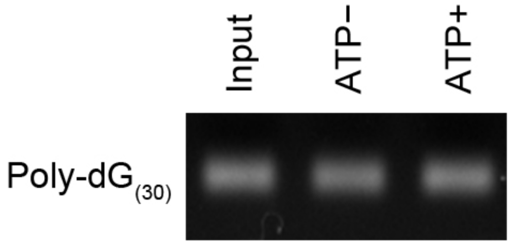

Figure S4. Poly-dG$_{(30)}$ was not degraded in the solution outside lysosomes. Isolated lysosomes were incubated for 5 min at 37°C in the presence or absence of an ATP regeneration system. The lysosomes were removed by centrifugation, and the solution outside lysosomes was incubated with 100 pmol of poly-dG$_{(30)}$ for 5 min at 37°C.

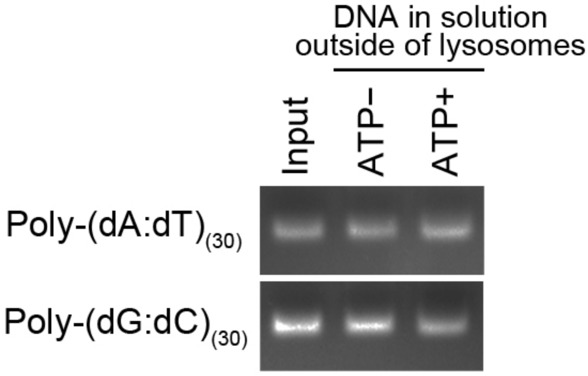

Figure S5. Poly-(dA:dT)$_{(30)}$ or poly-(dG:dC)$_{(30)}$ (100 pmol) were incubated with isolated lysosomes in the presence or absence of an ATP regeneration system. Then, levels of dsDNA remaining in the solution outside of lysosomes were analyzed by agarose gel electrophoresis.
